# EXPLORING THE IMPACT OF CHORAL SINGING ON CHANGE IN PERCEIVED DISTRESS FOR CARE PARTNERS OF PERSONS WITH DEMENTIA

**DOI:** 10.1093/geroni/igad104.2595

**Published:** 2023-12-21

**Authors:** Stuart MacDonald, Nicholas Tamburri, Cynthia McDowell, Andre Smith, Carren Dujela, Debra Sheets

**Affiliations:** University of Victoria, Victoria, British Columbia, Canada; University of Victoria, Victoria, British Columbia, Canada; University of Victoria, Victoria, British Columbia, Canada; University of Victoria, Victoria, British Columbia, Canada; University of Victoria, Victoria, British Columbia, Canada; University of Victoria, Victoria, British Columbia, Canada

## Abstract

Family care partners (CP) of persons with dementia (PwD) report high levels of psychological distress that impact many facets of their lives. Arts-based interventions have shown promise for improving psychological health. Here, we describe findings from the Voices in Motion (ViM) study, a weekly lifestyle intervention for CP-PwD dyads, with specific emphasis on examining the impact of choral singing on level and change in perceived distress for CP. Data from n=29 CP (Mage=67.7 years, 80% female) were used to examine change in caregiver distress, indexed using the Zarit Caregiver Burden Interview (ZBI). ViM employed an intensive longitudinal design characterized by monthly retests embedded within 3.5-month choral seasons (up to 7 assessments per person). Choral seasons were separated by a 4-month summer break, yielding a naturalistic ABA experimental design optimal for evaluating the impact of choral singing on caregiver distress. Multilevel models of change with piecewise splines were fit; REML was employed due to its advantageous properties for small samples. A significant reduction in caregiver distress (-1.14 units per month, p<.01) was observed across the 3.3-month span of season 1. Following the 4-month summer break, level of caregiver distress at the outset of season 2 had increased back to initial baseline levels (ZBI =19.9 units – a moderate to borderline-high level), thereby underscoring the importance of the choir intervention. These findings suggest that lifestyle engagement may offer an effective complement for lessening the impact of caregiver distress for CP, with implications for the development of dementia care plans.

